# Pathogenesis of MRSA–influenza co-infection: implications for ECMO therapeutic strategies – a mini-review

**DOI:** 10.3389/fimmu.2026.1741603

**Published:** 2026-04-10

**Authors:** WenFei Wang, Cong Liu, YaJun Li, YaWen Zheng, QingLi Dou

**Affiliations:** 1 Department of Emergency Medicine, The People’s Hospital of Baoan Shenzhen, Shenzhen, China; 2 Department of Emergency Intensive Care Unit (EICU), Shenzhen Baoan Air Sea Hospital, Shenzhen, China

**Keywords:** ARDS, ECMO, influenza, MRSA, necrotizing pneumonia, *Staphylococcus aureus*

## Abstract

Influenza virus (*Influenzavirus A*) and *Staphylococcus aureus* (*S. aureus*) co-infection is a critical clinical challenge, often leading to severe complications such as necrotizing pneumonia. This review elucidates the mechanisms by which influenza virus facilitates *S. aureus* infections through epithelial damage and immune modulation, thereby exacerbating pulmonary injury. Specifically, influenza virus infection damages respiratory epithelial cells and disrupts the integrity of the lung barrier. This process facilitates the invasion of *S. aureus*, which produces various virulence factors, including Panton-Valentine leukocidin (PVL) and phenol-soluble modulins (PSMs), leading to enhanced inflammation and tissue destruction. Furthermore, methicillin-resistant *S. aureus* (MRSA) strains are associated with higher morbidity and mortality due to their resistance to beta-lactam antibiotics and increased toxin production. Understanding the interplay between influenza virus-induced epithelial damage and *S. aureus* toxin-mediated immune responses is crucial for developing effective therapeutic interventions to mitigate the severity of necrotizing pneumonia. This review also explores the potential roles of adjunctive therapies, such as extracorporeal membrane oxygenation (ECMO) and novel agents like intravenous immunoglobulin (IVIG) and N-acetylcysteine (NAC), in redefining treatment paradigms for these severe infections.

## 1 Introduction

Influenza virus-induced respiratory tract infections represent a major global health burden, accounting for significant morbidity and mortality. However, co-infection with *Staphylococcus aureus* (*S. aureus*) substantially elevates the risk of developing severe complications, particularly necrotizing pneumonia (1). The emergence of methicillin-resistant *S. aureus* (MRSA) has further intensified clinical challenges. MRSA infections are associated with higher morbidity and mortality rates compared to methicillin-sensitive *S. aureus* (MSSA) infections. The resistance of MRSA to beta-lactam antibiotics poses significant treatment challenges, necessitating the use of alternative antimicrobial agents (2). Furthermore, MRSA isolates often exhibit increased production of virulence factors such as Panton-Valentine leukocidin (PVL) and phenol-soluble modulins (PSMs), which exacerbate tissue damage and immune dysregulation in the context of influenza co-infection (3).

The pathogenesis of necrotizing pneumonia involves complex interactions between *S. aureus*, particularly PVL-positive strains, and the host immune system (4). Despite recent advances, the precise molecular mechanisms underlying this process remain incompletely elucidated (5). The role of *S. aureus* toxins in this disease process is well-documented. PVL, a potent leukocidin produced by (2)specific strains of *S. aureus*, induces cytolysis of neutrophils, monocytes, and macrophages, thereby impairing the host’s immune response. Additionally, other staphylococcal components, such as PSMs, contribute to inflammation by disrupting host cell membrane integrity and promoting the release of pro-inflammatory mediators. These mechanisms collectively result in extensive lung tissue destruction, characterized by necrotizing pneumonia (6).

Given the complexity of these pathogenic processes, there is an urgent need to develop effective therapeutic strategies for the management of influenza-associated *S. aureus* infections. Adjunctive therapies, such as extracorporeal membrane oxygenation (ECMO) and novel therapeutic agents, show promise in modulating the immune response and mitigating tissue damage (7). While ECMO application in isolated respiratory failure is well-established per ELSO guidelines, the synergistic virulence of influenza-MRSA coinfection precipitates uniquely rapid cardiopulmonary deterioration. This review specifically examines ECMO’s efficacy in toxin-mediated ARDS—a scenario inadequately addressed in current guidelines and characterized by distinct immune dysregulation patterns demanding targeted therapeutic strategies beyond conventional ECMO protocols (8).

## 2 Methods

This narrative review synthesizes preclinical and clinical evidence on ECMO’s roles in MRSA and Influenza Virus Coinfection. We systematically searched PubMed and Embase databases (January 2009–June 2025) to include foundational ECMO studies from the H1N1 pandemic era. The search strategy employed the following terms: (“ECMO” OR “Extracorporeal Membrane Oxygenation”) AND (“MRSA” OR “Methicillin-Resistant Staphylococcus aureus”) AND (“Influenza Virus Coinfection”).

### 2.1 Inclusion and exclusion criteria

Studies were included if they specifically reported on adult patients with influenza A virus and MRSA coinfection who received ECMO support. References from studies that had their full text reviewed but did not meet inclusion criteria were searched to identify any missed literature, with resultant abstracts reviewed utilizing the same inclusion criteria above. Studies were excluded if they did not report on the MRSA and Influenza Virus Coinfection and ECMO support.

## 3 Methicillin-resistant *Staphylococcus aureus*

*Staphylococcus aureus* is a well-documented human pathogen capable of causing a wide range of diseases. It is firmly established that S. aureus secretes a variety of virulence factors that have evolved to evade the human immune system. Furthermore, there has been a notable increase in the incidence of *S. aureus* infections among previously healthy individuals (1). Clinical studies have shown that Panton-Valentine leukocidin (PVL)-positive *S. aureus* strains are almost exclusively associated with severe necrotizing pneumonia in otherwise healthy young patients, often following influenza-like illnesses. Conversely, PVL-negative strains typically cause milder, nonspecific pneumonia, predominantly in older adults (aged ≥60 years) (9).

Methicillin-sensitive *Staphylococcus aureus* (MSSA) and methicillin-resistant *S. aureus* (MRSA) produce an array of virulence factors that drive the pathogenesis of pulmonary infections. These factors can be broadly categorized into pathogen-associated molecular patterns (PAMPs) and secreted exotoxins (6). PAMPs encompass cell wall components such as lipoproteins, lipoteichoic acid, peptidoglycan, and protein A, which activate innate immunity through recognition by Toll-like receptors (TLRs) and other pattern recognition receptors. The exotoxin family includes alpha-hemolysin (Hla), Panton-Valentine leukocidin (PVL), and α-type phenol-soluble modulins (PSMα), with PVL being the most extensively characterized virulence factor in pulmonary infections (10). While these toxins engage distinct cellular receptors, they converge on common downstream pathways involving NF-κB signaling activation and subsequent release of pro-inflammatory mediators (11).

The pathophysiological cascade leading to necrotizing pneumonia involves a complex interplay between bacterial virulence and dysregulated host immunity. Following alveolar invasion, *S. aureus* initiates a dual-phase assault: (1) PAMP-mediated activation of TLR signaling induces massive neutrophil recruitment through CXCL8/IL-8 and other chemokine release (12, 13); (2) Subsequent PVL-dependent neutrophil lysis converts these innate immune cells from defenders into perpetrators of tissue destruction. As primary responders, neutrophils deploy an antimicrobial arsenal including reactive oxygen species (ROS), matrix metalloproteinases (MMPs), and cathelicidins - double-edged swords that eliminate pathogens at the cost of collateral tissue damage (14). PVL exacerbates this paradox through its bimodal action: Sublytic concentrations prime neutrophils for enhanced ROS production, while cytotoxic doses induce pore-mediated cell lysis (15). This results in catastrophic release of neutrophil-derived elastase, myeloperoxidase, and extracellular traps (NETs), which collectively degrade alveolar epithelial tight junctions and endothelial basement membranes (16). The toxin-mediated nature of this pathology has critical therapeutic implications. Antimicrobial agents cannot effectively neutralize pre-formed toxins or reverse established tissue damage (17). This pathophysiological understanding underscores the necessity for early diagnosis and prompt initiation of toxin-suppressive therapies in conjunction with bactericidal antibiotics.

## 4 Coinfection of influenza virus and MRSA

Influenza viruses belong to the Orthomyxoviridae family and are classified into types A, B, and C. Influenza A viruses are further subdivided based on the antigenic characteristics of their surface hemagglutinin (H) and neuraminidase (N) glycoproteins. Among the 15 H and 9 N subtypes, only H1, H2, H3, N1, and N2 have caused extensive human outbreaks (18). The pandemic H1N1 influenza A virus (influenza A [H1N1] pdm09 virus) emerged as a result of genetic reassortment, leading to a novel strain with distinct antigens (19). Initial reports of severe pandemic A(H1N1)2009 influenza virus infection described a predominantly viral pneumonitis, with bacterial coinfections being relatively uncommon (20). In cases of H1N1 influenza with bacterial coinfection, *Staphylococcus aureus* was the most frequently identified pathogen in respiratory secretions, followed by Pseudomonas species, Streptococcus pneumoniae, Haemophilus influenzae, and Streptococcus pyogenes. Among *S. aureus* isolates, 48% were MRSA (5). Although rare, the epidemiology of PVL *S. aureus* (PVL-SA) pneumonia as a complication of influenza coinfection, particularly in young adults, remains poorly understood (21). Notably, Lina et al. (1999) observed that PVL-positive necrotizing pneumonia often occurred in patients with a preceding viral infection (22). The typical presentation of MRSA coinfection following pH1N1 influenza is characterized by severe pneumonia with hemoptysis, hypotension, rapid progression to septic shock, and respiratory failure requiring ventilatory support. MRSA-induced necrotizing pneumonia is associated with a high fatality rate (5, 23). The synergy between influenza virus and *S. aureus* significantly complicates the pathogenesis of necrotizing pneumonia.

The influenza virus exacerbates the inflammatory potential of *S. aureus* toxins by amplifying monocyte-derived chemokine production, thereby enhancing pulmonary infiltration of neutrophils, monocytes, and macrophages (24). This synergistic effect aligns with the recognized pattern of viral infections, particularly influenza, which trigger epithelial cell activation through cytokine/chemokine induction. It is a crucial mechanism facilitating neutrophil recruitment to infection sites (25). Notably, while viral infection alone did not induce detectable epithelial cell death in experimental models, PVL toxin demonstrated distinct cytotoxic effects. Specifically, PVL induced neutrophil apoptosis and subsequent epithelial damage, with additional inflammatory contributions from staphylococcal virulence factors such as phenol-soluble modulins (PSMs) (26).

Influenza virus infection potentiates neutrophil-mediated proinflammatory responses through synergistic interactions with *Staphylococcus aureus* virulence factors. The combined action of influenza virus and *S. aureus* toxins induces synergistic activation of human monocytes, correlating with enhanced α-toxin (Hla) cytotoxicity toward influenza-primed monocytes (27). This augmented inflammatory potential drives neutrophil extravasation and amplifies their accumulation and necroptosis at infection sites, creating a pathogenic feedback loop. Clinically, this interplay explains the disproportionate association of MRSA with severe pneumonia outcomes, as MRSA strains overexpress PSMs, Hla, and PVL compared to MSSA (27). Notably, PVL’s proinflammatory and cytotoxic effects on neutrophils are substantially augmented by antecedent influenza infection. While the mechanisms underlying influenza-induced susceptibility to *S. aureus* superinfection remain incompletely characterized, post-influenza pneumonia models implicate immune-mediated tissue damage as a key driver. Specifically, overzealous immune responses—marked by excessive leukocyte infiltration into pulmonary tissues—trigger cellular destruction and exacerbate respiratory tract pathology (28). The initial recruitment of monocytes and macrophages to alveolar spaces serves as a critical first-line defense, but dysregulated amplification of this process culminates in life-threatening pulmonary complications. Multiple synergistic mechanisms have been proposed to explain the pathogenesis: Influenza-induced damage to airway epithelial cells facilitates bacterial invasion by compromising mucosal integrity; impaired bacterial clearance by alveolar macrophages, coupled with influenza-mediated downregulation of Toll-like receptor signaling; Type I interferon (IFN-α/β)-dependent suppression of neutrophil recruitment, creating permissive conditions for secondary infection (29) (**Table 1**).

**Table 1:** Pathophysiological timeline and critical intervention windows in influenza-MRSA coinfection.

Stage	Core Pathogenesis	Clinical Markers & Temporal Patterns	ECMO Implications
Viral Phase (0-72h)	• Influenza HA-mediated epithelial syncytia formation • TLR3/7/8-induced type I IFN storm	• Acute fever (>39.5°C), myalgia • Peak nasopharyngeal viral load: 24-48h ⁠(18, 19)	Antiviral window (NAI initiation <48h)
Bacterial Colonization >(24-96h)	• Increase 300% integrin β1 expression • NA-exposed collagen binding • MRSA ClfB-fibrinogen interaction	• Increase 78% MRSA+ sputum cultures after 48h ⁠(5, 23) • PVL-PCR+ predicts necrotizing pneumonia (OR=6.7; 95%CI 3.1-14.2)	CRBSI prophylaxis (MRSA-covering lock therapy)
Toxin-Mediated Injury (72-120h)	• PVL-induced neutrophil lysis • NETosis peak at 96h ⁠(14, 16) • Hla-mediated tight junction disruption	• Lactate >4.0 mmol/L predicts MOF ⁠(27) • Radiological: rapid cavitation within 72h	Anticoagulation adjustment (Target anti-Xa 0.3-0.7 IU/mL in sepsis)
Immunoparalysis (>120h)	• HLA-DR expression <30% • Treg expansion (CD4+CD25+FoxP3+) • Increase IL-10 5-fold	• Increase 41% fungal superinfection • 61% mortality in immunocompromised ECMO ⁠(30, 31)	Weaning stratification (Incorporating lymphopenia in scores)

## 5 ECMO therapy principles and clinical implementation

Three classes of antivirals target influenza: neuraminidase inhibitors (zanamivir, oseltamivir, peramivir, laninamivir), adamantanes (amantadine, rimantadine), and endonuclease inhibitors (baloxavir) (32). Cross-resistance between oseltamivir and peramivir arises from structural similarity, with the H275Y neuraminidase mutation inducing steric resistance to both. Zanamivir retains efficacy against H275Y variants, while E119D/G mutations drive pan-neuraminidase inhibitor resistance in pandemic H1N1 strains (33). Adamantane resistance stems from M2 protein transmembrane mutations, disabling amantadine/rimantadine activity (34). Standardized guidelines for newer agents remain urgently needed.

Adjunctive therapeutic strategies for influenza—including extracorporeal membrane oxygenation (ECMO), N-acetylcysteine (NAC), intravenous immunoglobulin (IVIG), and glucocorticoids—remain under investigation. For ECMO specifically, evidence-based selection criteria emphasize application in severe ARDS failing conventional ventilation, with age <65 years and single-organ failure as favorable prognostic factors according to ELSO registry analyses (35). Current evidence demonstrates no mortality benefit from glucocorticoids in severe influenza, with potential harm due to immunosuppression (36). In immunocompromised hosts (ICH), corticosteroids may exacerbate co-infections by impairing innate and adaptive immunity. However, isolated reports suggest glucocorticoid efficacy in organizing pneumonia, postviral pneumonitis, and H1N1 pneumonia during pregnancy (37, 38). Clinical practice demonstrates considerable variability, largely due to ongoing debates regarding the roles of adjunctive therapies. Regarding anticoagulation, sepsis-induced coagulopathy necessitates circuit-specific protocols: ELSO recommends targeting anti-Xa levels of 0.3–0.7 IU/mL or aPTT 40–60s given platelet dysfunction and heparin resistance in MRSA-influenza coinfections (39). Emerging treatments, such as ECMO and novel agents like IVIG and NAC, hold promise to redefine treatment paradigms, pending validation in robust clinical trials.

ECMO therapy represents a potential adjunct or rescue therapy for severe H1N1 pneumonia complicated by respiratory failure; clinical decisions on modality selection should be guided by hemodynamic status: Veno-Venous (VV) ECMO predominates in isolated respiratory failure (81% in ELSO H1N1 cohort), while veno-arterial (VA) ECMO is reserved for cardiopulmonary collapse (8). The optimal cannulation window remains critical—retrospective data indicate survival advantage when initiated within 7 days of intubation (adjusted OR 2.1; 95% CI 1.3–3.6) (40). However, definitive conclusions remain limited due to the absence of randomized controlled trials. ECMO support has been associated with prolonged mechanical ventilation duration, extended ICU stays, and high mortality rates. Notably, ICH populations with H1N1 infection complicated by acute respiratory distress syndrome (ARDS) who underwent ECMO demonstrated mortality rates as high as 61%, exceeding the 42% overall rate in non-immunocompromised adults per 2022 ELSO report (30). In a comparative study of MRSA and MSSA patients, both groups exhibited comparable durations of ECMO support and similar intubation-to-ECMO intervals (41). Early initiation of ECMO following mechanical ventilation has been associated with improved survival outcomes (42).

**Figure 1:**
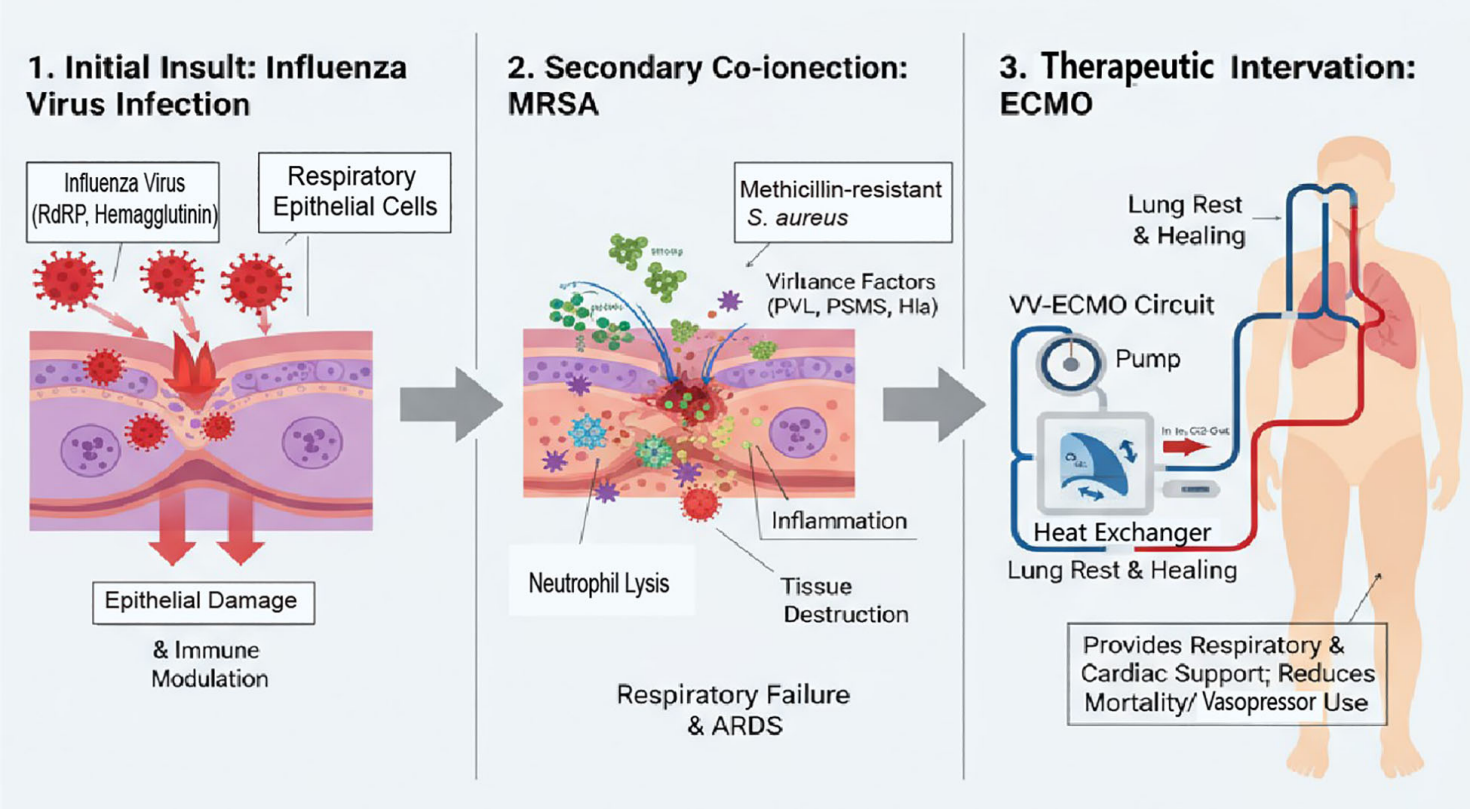
Pathophysiologica cascade and ECMO intervention in co-infected patients.

Among patients with ARDS of diverse etiologies, VV ECMO has shown clinical applicability. The novel cANNULATION score (incorporating pH, lactate, and pressor requirements) may optimize timing decisions with 78% predictive accuracy for successful decannulation (43). Despite the persistent complications commonly associated with this modality, a noTable 63% of patients achieved ARDS recovery and successful ECMO weaning, with 49% ultimately discharged from the ICU (44). Sepsis management during ECMO requires multidisciplinary protocols: citrate-based regional anticoagulation demonstrates 31% lower hemorrhage rates compared to systemic heparinization in septic ECMO patients (45). However, VV ECMO remains burdened by significant complications, predominantly sepsis, multiorgan failure, and hemorrhagic events. The extent to which such complications can be mitigated through advancements in ECMO technology or adjunctive supportive therapies, as opposed to being inherently linked to the critical condition of ECMO-treated patients, remains unclear (46). ELSO registry data reveal VV-ECMO survival stratification by etiology: influenza-associated ARDS survival (63%) exceeds bacterial pneumonia (48%), but MRSA coinfection reduces survival to 39% (31).A systematic review of ECMO-treated H1N1 influenza patients demonstrated that the majority underwent VV ECMO. When compared to VA ECMO, VV ECMO yielded comparable mortality rates(**Figure 1**) (47, 48).

## 6 Limitations

The interpretation of ECMO efficacy remains provisionally guided by observational data due to the absence of randomized trials. Significant heterogeneity exists across studies in patient populations and management protocols. Inherent biases include preferential ECMO utilization at high-volume centers and overrepresentation of post-2017 technological outcomes. These constraints necessitate cautious clinical extrapolation.

## 7 Conclusion

The interaction between influenza virus and *Staphylococcus aureus* in the pathogenesis of necrotizing pneumonia is a complex process involving epithelial damage, immune dysregulation, and bacterial toxin-mediated tissue destruction. While significant progress has been made in understanding these mechanisms, there remains an urgent need for effective therapeutic strategies to manage these severe infections. Emerging therapies, such as ECMO and novel immunomodulatory agents, hold promise in redefining treatment paradigms. However, the validation of these approaches through robust clinical trials is essential to establish evidence-based guidelines for the management of influenza-associated *S. aureus* infections.
